# Enzymatic modifications of exopolysaccharides enhance bacterial persistence

**DOI:** 10.3389/fmicb.2015.00471

**Published:** 2015-05-15

**Authors:** Gregory B. Whitfield, Lindsey S. Marmont, P. Lynne Howell

**Affiliations:** ^1^Program in Molecular Structure and Function, Research Institute, The Hospital for Sick ChildrenToronto, ON, Canada; ^2^Department of Biochemistry, Faculty of Medicine, University of TorontoToronto, ON, Canada

**Keywords:** Biofilm, exopolysaccharide, PNAG, PIA, alginate, PEL, VPS, cepacian

## Abstract

Biofilms are surface-attached communities of bacterial cells embedded in a self-produced matrix that are found ubiquitously in nature. The biofilm matrix is composed of various extracellular polymeric substances, which confer advantages to the encapsulated bacteria by protecting them from eradication. The matrix composition varies between species and is dependent on the environmental niche that the bacteria inhabit. Exopolysaccharides (EPS) play a variety of important roles in biofilm formation in numerous bacterial species. The ability of bacteria to thrive in a broad range of environmental settings is reflected in part by the structural diversity of the EPS produced both within individual bacterial strains as well as by different species. This variability is achieved through polymerization of distinct sugar moieties into homo- or hetero-polymers, as well as post-polymerization modification of the polysaccharide. Specific enzymes that are unique to the production of each polymer can transfer or remove non-carbohydrate moieties, or in other cases, epimerize the sugar units. These modifications alter the physicochemical properties of the polymer, which in turn can affect bacterial pathogenicity, virulence, and environmental adaptability. Herein, we review the diversity of modifications that the EPS alginate, the Pel polysaccharide, *Vibrio* polysaccharide, cepacian, glycosaminoglycans, and poly-*N*-acetyl-glucosamine undergo during biosynthesis. These are EPS produced by human pathogenic bacteria for which studies have begun to unravel the effect modifications have on their physicochemical and biological properties. The biological advantages these polymer modifications confer to the bacteria that produce them will be discussed. The expanding list of identified modifications will allow future efforts to focus on linking these modifications to specific biosynthetic genes and biofilm phenotypes.

## Introduction

Bacteria faced with fluctuating or stressful environmental conditions can undergo a number of physiological changes. One common tactic that bacteria use to adapt to their surroundings is to grow as a multicellular community or biofilm. Biofilm formation begins with attachment of the bacteria to a surface or, in the case of some infectious biofilms, embedding of the bacteria in host-derived tissue or mucous. This is followed by bacterial aggregation, colony development, and the secretion of self-produced polymeric substances, which form a matrix that encapsulates and protects the bacteria (Ohman, 1986; Allesen-Holm et al., 2006). This matrix is composed of nucleic acids, proteins, lipids, and extracellular polysaccharides (EPS; Flemming and Wingender, 2010), with the types and ratio of each component varying between bacterial species and environmental conditions. Once the biofilm has matured into a robust structure it becomes exceedingly difficult to eradicate, and is typically capable of enduring mechanical, biological, and chemical means of elimination. Bacteria form biofilms in nearly all environments studied to date (Bjarnsholt et al., 2013b), and are implicated in the contamination of surfaces as diverse as the International Space Station (Kim et al., 2013), ship hulls (Schultz et al., 2011), and oil storage and transfer infrastructure (Lenhart et al., 2014). Biofilms are also of major concern in medical settings, where they are responsible for the chronic infection of burn wounds, eye and skin lacerations, and pneumonia in CF patients (Lyczak et al., 2000). The contamination of medical devices such as catheters, prosthetic joints, and ventilators (Veerachamy et al., 2014) has also been well documented. In these environments EPS often contribute to the formation, growth, and preservation of biofilm architecture and also serve to protect the bacteria against antibiotics, desiccation, and the host’s immune defenses.

**FIGURE 1:**
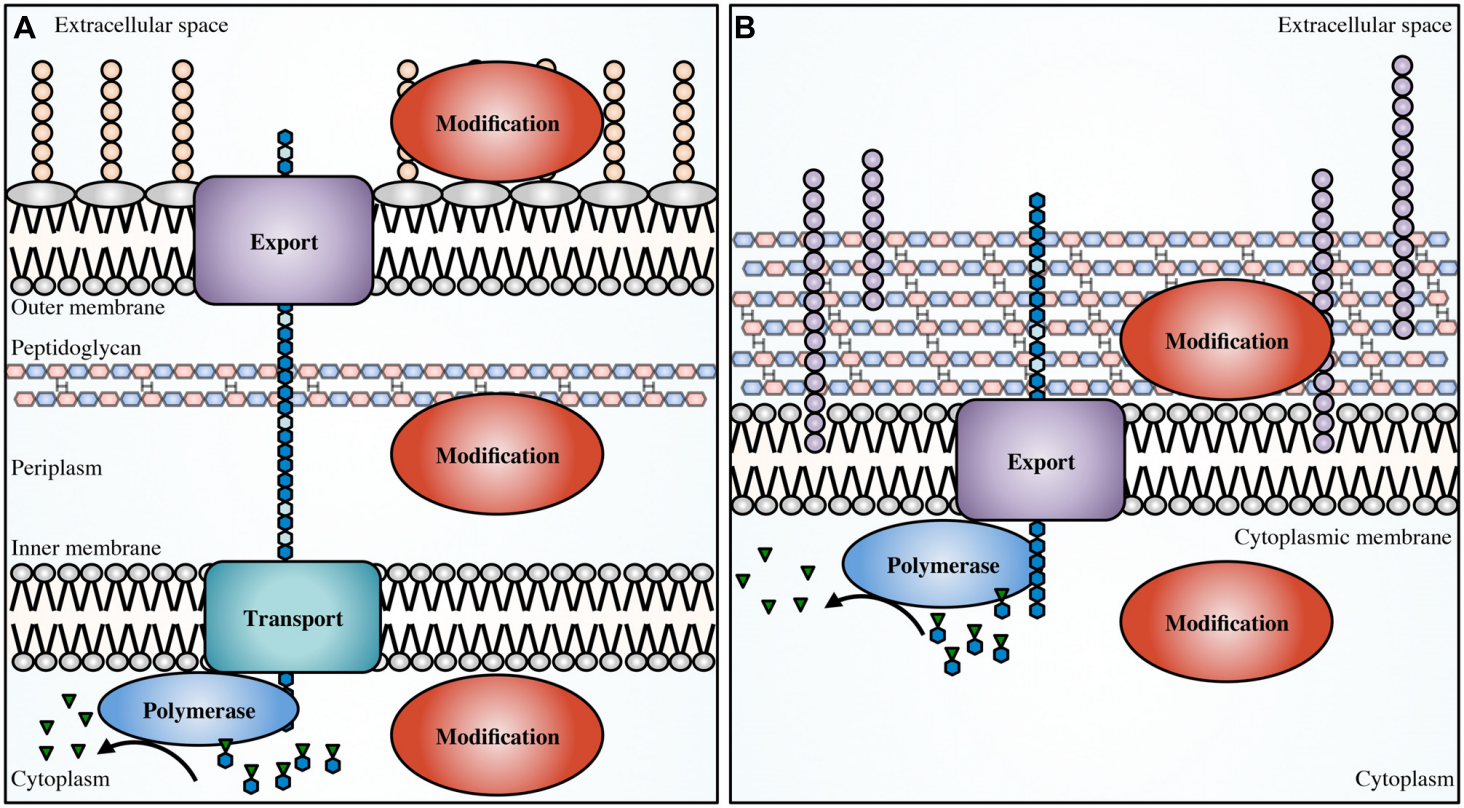
**Generalized EPS biosynthetic platforms.** *Not to scale.* In Gram-negative bacteria **(A)** modifications to the polymer can occur in the cytoplasm, periplasm and in the extracellular space. In Gram-positive bacteria **(B)** modifications can occur in the cytoplasm and extracellular space. Polymer biosynthetic systems are represented here as follows: activated sugars (blue hexagons with green inverted triangles) are assembled by a polymerase (blue), transported across the inner membrane (teal; polymerization and transport may be coupled and performed by a single protein), and exported (purple) across the outer membrane in Gram-negative bacteria, or exported across the cytoplasmic membrane in Gram-positive bacteria. Modifications can be performed in any of these cellular compartments (red). Also shown, LPS (tan circles), teichoic acids (light purple circles), and EPS (blue hexagons).

Biosynthesis of EPS begins in the cytoplasm with the generation of activated precursor sugars. These precursors are often taken from common cellular sugar pools and are modified for specific use in EPS biosynthesis pathways, prior to polymerization (**Figure 1**). In Gram-negative bacteria, the polymer is transported across the inner membrane to the periplasm during synthesis; whereas in Gram-positive bacteria the polymer is transported directly to the extracellular space. Modifications to the polymer can occur in the cytoplasm (Atkin et al., 2014) or the periplasm (Colvin et al., 2013; Baker et al., 2014; Little et al., 2014b; Wolfram et al., 2014) prior to export across the outer membrane, and in the extracellular space in both Gram-positive and Gram-negative bacteria (Rozeboom et al., 2008; Little et al., 2014a).

The chemical structure of EPS from different bacterial species, or even within the same organism, can vary greatly. Bacterial EPS are usually composed of hexose sugars, but pentose sugars have also been identified. *Rhizobium huakuii* EPS contains ribose (Hisamatsu et al., 1997), while some marine bacteria produce EPS with xylose and ribose moieties (Kwon et al., 2002). EPS can be homo- or hetero-polymers, and have branching side chains (Byrd et al., 2009; Cescutti et al., 2010) or be simple linear sugar polymers (Linker and Jones, 1966; Maira-Litràn et al., 2002). They can be as short as dimers and trimers, or thousands of saccharide repeat units long (González et al., 1998), depending on the mechanisms of chain length regulation, and can even be woven together to form fibers (Benziman et al., 1980).

**FIGURE 2:**
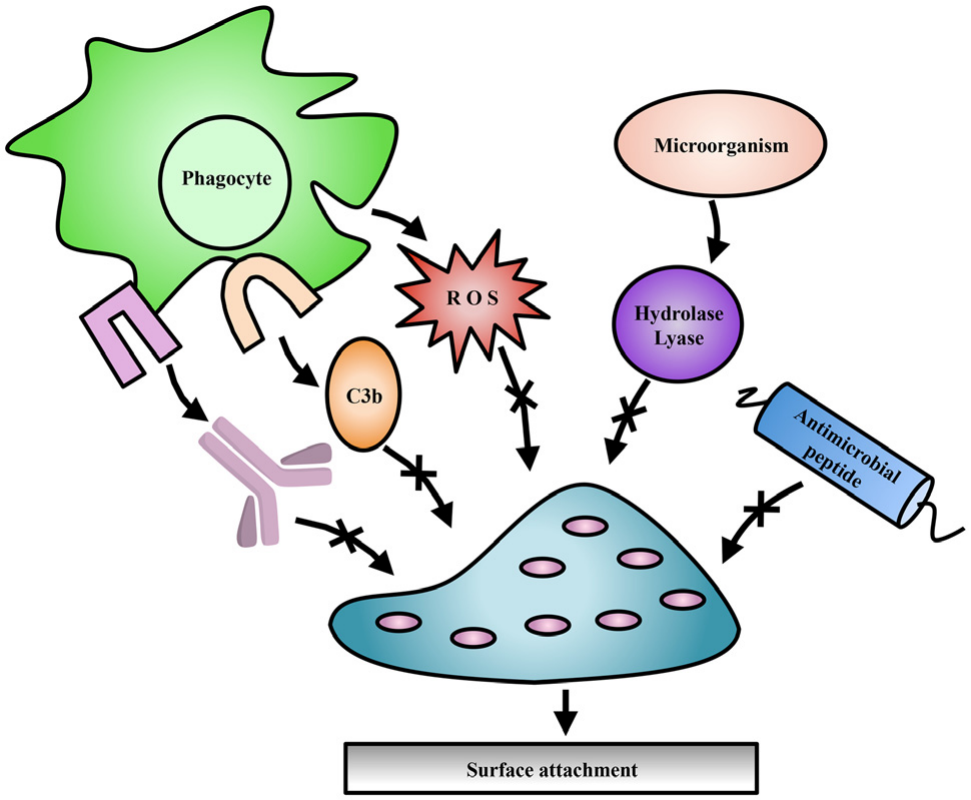
**Exopolysaccharide modifications offer protection to bacteria.** Modifications to EPS contribute to evasion of host immune mechanisms such as complement deposition (C3b), and specific antibody production (lavender). Modifications have also been shown to protect against ROS produced by immune cells, antimicrobial peptides, and EPS degradation enzymes produced by competing microorganisms.

Exopolysaccharides can be modified by the action of transferases and hydrolases which add or remove functional groups such as acetyls, pyruvyls (Marzocca et al., 1991), glyceryls (Kuo et al., 1986), succinyls (Reuber and Walker, 1993), lactyls (Maalej et al., 2014), or a combination of these, leading to variations in polymer surface electrostatics and solubility. Additionally, epimerization can drastically alter the structural conformation of polysaccharides, affecting polymer interactions within the biofilm (Steigedal et al., 2008). Some of these modifications have been studied with respect to their importance in bacterial virulence, pathogenesis, biofilm formation, or symbiosis (**Figure 2**; Ridout et al., 1997), as well as their commercial utility in the food and cosmetic industries.

Despite this wealth of knowledge, there remain a number of unresolved questions regarding the biological implications of EPS modifications. In this review, we explore the modifications that biofilm-forming EPS produced by human pathogenic bacteria undergo and discuss the proteins involved in modification, as well as the role modifications play in bacterial persistence in the environment and host.

## Alginate

**FIGURE 3:**
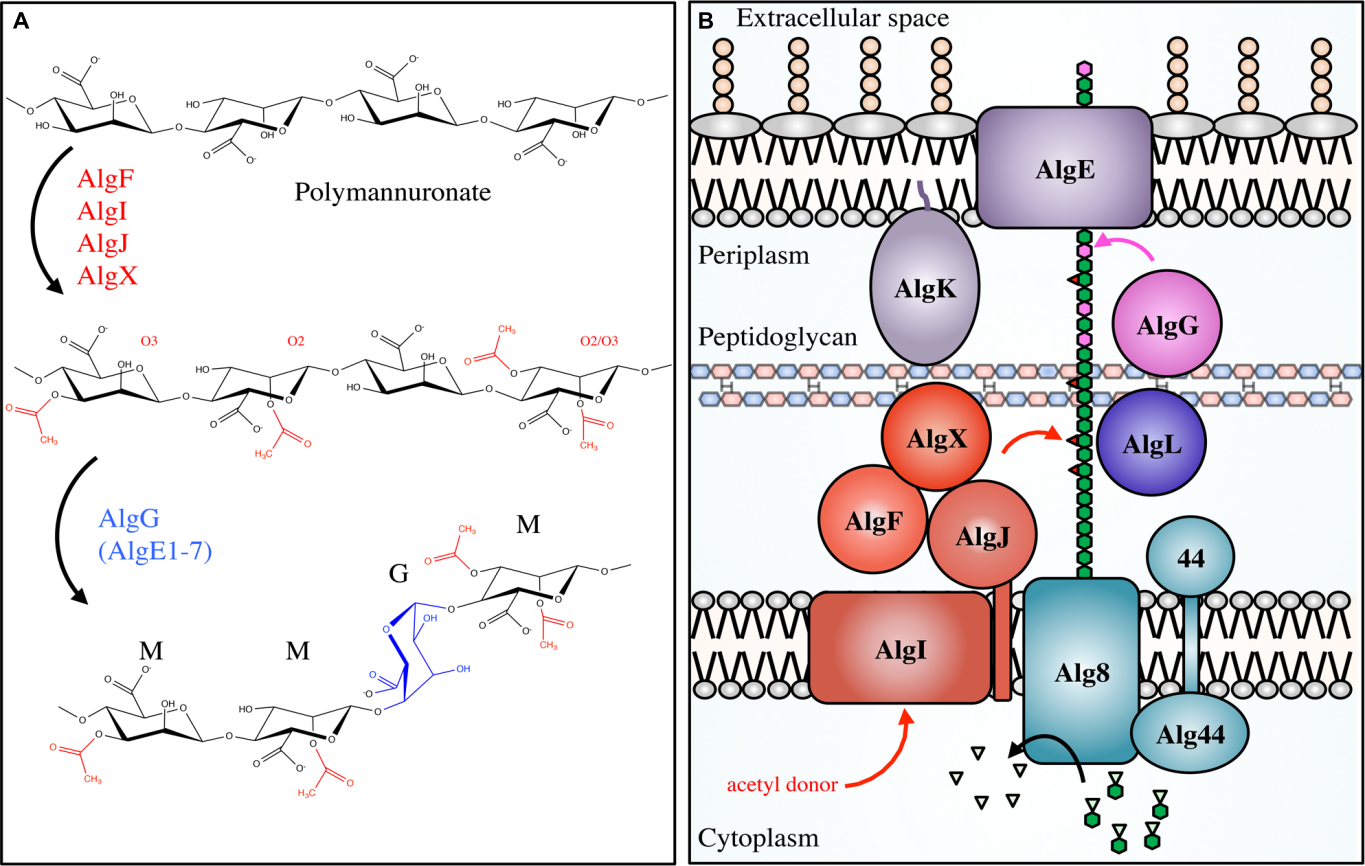
***Pseudomonal* alginate modifications and biosynthetic apparatus.** *Not to scale.* **(A)** Alginate is first polymerized as β-scD-(1→4)-polyM (top) before being acetylated at the O2 and/or O3 positions by the concerted actions of AlgIJFX. Acetylated ManA residues (middle) cannot be epimerized to L-GulA by AlgG (bottom). **(B)** The proteins involved in alginate biosynthesis in *Pseudomonas aeruginosa*. PolyM (green hexagon chain) is synthesized in the cytoplasm from the nucleotide-sugar precursor GDP-ManA (green hexagons with inverted triangles). It is polymerized, and transported across the inner membrane via Alg8/44, acetylated (red triangles) by the concerted action of AlgFIJX, and epimerized to GulA (magenta) by AlgG. AlgL possesses lyase activity; AlgK is a lipoprotein required for efficient localization of AlgE, the porin required for alginate export.

Alginate synthesis has been characterized in several species of brown algae, as well as in the genera *Azotobacter* and *Pseudomonas* (Gorin and Spencer, 1966; Evans and Linker, 1973; Govan et al., 1981; Gacesa, 1988). Bacterial alginate is a high molecular weight, linear polysaccharide composed of β-1,4-linked D-ManA and variable amounts of its C5 epimer L-GulA (Smidsrød and Draget, 1996). In *Pseudomonas aeruginosa* and *Azotobacter vinelandii*, alginate is initially synthesized as polyM in the cytoplasm and is shuttled across the inner membrane to the periplasm, where it is randomly acetylated at the O2 and/or O3 hydroxyl positions (**Table 1**; **Figure 3**; Franklin and Ohman, 1993, 1996, 2002). ManA residues that are not acetylated serve as substrates for epimerization at the C5 position by the enzyme AlgG in the periplasm, leading to the formation of mixed ManA and GulA segments (MG-blocks) as well as non-epimerized sections (M-blocks; Gacesa, 1988; Jain et al., 2003).

**Table 1:** Exopolysaccharide modifying enzymes of pathogenic bacteria.

Protein name	GenBank Accession number	Organism(s) studied^a^	Function	Cellular localization	PDB code^b^	Additional comments	Reference
AlgG	NP_252235.1 AAP46694.1 YP_002798298.1	*P. aeruginosa* *P. fluorescens* *A. vinelandii*	Mannuronan C5-epimerase; introduces MG-blocks into polyM alginate	Periplasm	**4NK6**	Required for alginate production and epimerization in *P. aeruginosa* and *P. fluorescens*; cannot epimerize acetylated alginate	Chitnis and Ohman (1990), Franklin et al. (1994), Rehm et al. (1996), Morea et al. (2001), Gimmestad et al. (2003), Jain et al. (2003), Wolfram et al. (2014)
AlgE1 AlgE2 AlgE3 AlgE4 AlgE5 AlgE6 AlgE7	YP_002802178 YP_002802177 YP_002802176 YP_002802179 YP_002800496 YP_002802180 YP_002802182	*A. vinelandii*	Mannuronan C5-epimerases; alginate lyase (AlgE2, AlgE7); varying MG- and G-block forming activities	Extracellular	**AlgE4: 2PYG (active domain), 2AGM (regulatory domain) AlgE6:** **2ML2 and 2ML3 (regulatory domains)**	Ca^2+^-dependent; modular architecture composed of one or more epimerase active domains and activity enhancing regulatory domains	Ertesvåg et al. (1994, 1995, 1998), Ertesvåg and Valla (1999), Svanem et al. (1999, 2001), Aachmann et al. (2006), Rozeboom et al. (2008), Buchinger et al. (2014)
AlgJ	NP_252239.1	*P. aeruginosa*	Exhibits *in vitro* acetylesterase activity	Periplasm - IM tethered	**4O8V**	Required for alginate acetylation	Franklin and Ohman (1996, 2002), Baker et al. (2014)
AlgF	NP_252240.1 YP_002798293.1	*P. aeruginosa* *A. vinelandii*	Acetylation; specific role unknown	Periplasm	ND	Required for alginate acetylation	Franklin and Ohman (1993), Shinabarger et al. (1993), Vazquez et al. (1999), Franklin and Ohman (2002)
AlgI	NP_252238.1	*P. aeruginosa*	Acetylation; predicted MBOAT	Inner membrane	ND	Required for alginate acetylation	Franklin and Ohman (1996, 2002), Franklin et al. (2004)
AlgX	NP_252236.1	*P. aeruginosa* *A. vinelandii*	Acetylation; exhibits *in vitro* acetylesterase activity; terminal alginate acetylase	Periplasm	**4KNC**	Required for alginate production and acetylation	Monday and Schiller (1996), Robles-Price et al. (2004), Riley et al. (2013), Baker et al. (2014)
PelA	NP_251754.1	*P. aeruginosa*	Deacetylation; *in vitro* deacetylase activity	Periplasm	2VYO (30%; 511-794)	Modeled region of deacetylase, also has N-terminal hydrolase domain	Colvin et al. (2013)
BceO BceS BceU	YP_001116903 YP_001116907 YP_001116910	*B. cepacia* complex	Acetyltransferase	Inner membrane (predicted)	ND	Deletion of *bceS* – reduced acetylation	Ferreira et al. (2010)
VpsC VpsG	NP_230566.1 NP_230570.1	*V. cholerae*	Acetyltransferase (proposed)	ND	1T3D (92%; 2-172) 1T3D (97%; 1-140)	Modeled protein is an acetyltransferase	Fong et al. (2010)
PgaB	NP_415542.1	*E. coli*	Deacetylase; C-terminal carbohydrate binding module facilitates PNAG export	Outer membrane lipoprotein	**4F9J/4F9D**	Low catalytic efficiency allows for partial PNAG deacetylation	Wang et al. (2004), Itoh et al. (2008), Little et al. (2012, 2014b)
HmsF	NP_415542.1	*Y. pestis*	Deacetylase	Outer membrane	4F9D (88%; 43-646)	Modeled on PgaB	Forman et al. (2006)
IcaB	AAC06118.1 AAD52057.1	*S. epidermidis* *S. aureus*	Deacetylase	Extracellular	**4WCJ**	Low catalytic efficiency allows for partial PIA deacetylation	Pokrovskaya et al. (2013), Little et al. (2014a)
IcaC	AAC06119.1 AAD52058.1	*S. epidermidis* *S. aureus*	Succinyltransferase (proposed)	Membrane embedded	ND	Not experimentally determined	Atkin et al. (2014)

^a^Genus abbreviations as follows: *P. aeruginosa, Pseudomonas aeruginosa*; *P. fluorescens, Pseudomonas fluorescens*; *A. vinelandii, Azotobacter vinelandii*; *B. cepacia* complex, *Bcc*; *V. cholerae, Vibrio cholerae*; *E. coli, Escherichia coli*; *Y. pestis, Yersinia pestis*; *S. epidermidis, Staphylococcus epidermidis*; *S. aureus, Staphylococcus aureus*.

^b^Accession code for structure or structurally related protein, bold letters indicate that the structure has been experimentally determined. Where applicable, protein coverage (%) and residues modeled are listed in brackets. ND: not determined, Phyre^2^ (Kelley and Sternberg, 2009) was unable to model the protein.

*Pseudomonas aeruginosa* chronic lung infections in CF patients are the leading cause of morbidity and mortality. In these infections the production of alginate is often linked to poorer patient prognosis (Lyczak et al., 2000). In the lung, the clinical isolate *P. aeruginosa* FRD1 displays an alginate-overproducing, or mucoid phenotype due to mutations in negative regulatory elements, providing *P. aeruginosa* with the capability of adhering to respiratory tract epithelial cells and mucin (Marcus and Baker, 1985; Doig et al., 1987; Ramphal et al., 1987). In addition, alginate production has been linked to the hindrance of host cell-mediated phagocytosis and neutralization of ROS (Learn et al., 1987; Mai et al., 1993; Pier et al., 2001). Mucoid conversion is still not well understood, but *in vitro* experimentation has shown that nutrient and aeration levels (Buckmire, 1984; Krieg et al., 1986; Speert et al., 1990) and external stressors (Terry et al., 1991; Damron et al., 2011; Limoli et al., 2014) can induce mucoidy.

*Azotobacter vinelandii* is a soil-borne bacterium that is often used as a model organism for nitrogen fixation studies (Bulen et al., 1964). Although *A. vinelandii* is not a human pathogen, understanding the process of alginate biosynthesis in this organism has provided valuable insight into the biological significance of alginate production by *P. aeruginosa*. In *A. vinelandii,* alginate plays a unique and essential role where, under conditions of nutrient and environmental stress such as nitrogen starvation, *A. vinelandii* converts from a vegetative cell to a dormant cyst (Socolofsky and Wyss, 1961). Cyst development proceeds through deposition of a protective extracellular material composed primarily of alginate. The cyst layers are rich in proteins, lipids and carbohydrates, with the exine (outer layer) and intine (inner layer) containing carbohydrate material consisting of approximately 40 and 72% polyuronic acids, respectively (Lin and Sadoff, 1969). Cyst formation, much like the biofilm matrix, protects *A. vinelandii* from desiccation, and only when environmental conditions become more favorable will *A. vinelandii* convert back to the vegetative state by degrading the alginate barrier. In contrast to *P. aeruginosa*, *A. vinelandii* contains seven additional extracellular epimerases (AlgE1-7), capable of generating polyguluronate segments (G-blocks) in addition to MG-blocks (**Table 1**; Fischer and Dorfel, 1955; Haug et al., 1966, 1967). Despite the importance of alginate in cyst formation (Campos et al., 1996), alginate production by *A. vinelandii* has been studied primarily for its potential use as an alternative source of commercial alginate in place of traditional seaweed harvesting approaches.

Given the importance of alginate for the virulence of *P. aeruginosa* in the CF lung, the protective characteristics of *A. vinelandii* cysts, and the use of bacterial alginate as a convenient substitute for commercial eukaryotic alginate sources, there has been a drive to understand the consequences of alginate acetylation and epimerization on these processes.

## Alginate Acetylation

Alginate acetylation in *P. aeruginosa* requires the collective actions of the proteins AlgF, AlgI, AlgJ, and AlgX for the addition of acetyl groups to the O2 and/or O3 hydroxyl positions of polyM in the periplasm prior to epimerization and export (**Table 1**; **Figure 3**). Specifically, it is thought that AlgI transfers an acetyl group from an as yet unidentified cytoplasmic donor to AlgJ or AlgX, where it may be passed between them before transfer to the polymer by AlgX (Riley et al., 2013; Baker et al., 2014). AlgF is an important part of the acetylation process but has not yet been assigned a role. Based on the peptidoglycan *O*-acetylation machinery (Moynihan and Clarke, 2011), it seems that the presence of a MBOAT for acetyl-donor transport (AlgI), and an acetyltransferase (AlgX) should be sufficient for polysaccharide *O*-acetylation. As acetylation occurs at both the O2 and O3 positions, one hypothesis is that the proteins AlgJ and AlgF govern specificity. Although they have not yet been shown to directly bind alginate, they may still be able to regulate the position and frequency of acetyl addition under different environmental conditions. Genetic and biochemical experiments targeting the degree of alginate acetylation in combination with structural data may provide insight into these questions.

One of the purposes of EPS production is to serve as a structural component of the biofilm matrix. Alginate lacking *O*-acetyl groups has been analyzed for its ability to form structured biofilms using an acetylation defective *P. aeruginosa* FRD1 mutant. These studies revealed that the mutant was only able to produce small, unstructured microcolonies that sparsely populated the examined surface, suggesting an attachment defect (**Table 2**; Nivens et al., 2001). In contrast, FRD1 formed extensive biofilm structures that exhibited significant structural heterogeneity. In a separate study, an aggregation defect was revealed when the capacity for an acetylation-deficient FRD1 mutant to adhere to a steel surface was tested (Tielen et al., 2005). Additionally, the viscosity of extracellular material from the acetylation-defective mutant was significantly reduced in comparison to FRD1, suggesting that the loss of *O*-acetyl groups led to weakening of inter- and intra-polymer interactions within the biofilm matrix. This is supported by rheological studies of FRD1 biofilms, which suggested that inter-chain alginate interactions occur primarily through physical entanglements (Wloka et al., 2005). These entanglements supported an elastic biofilm architecture, which differed from *O*-acetylation-defective FRD1 mutants which produced weaker biofilms with reduced resistance to tensile forces. Based on these results, it was suggested that *O*-acetyl groups in alginate act as molecular hooks that improve the resistance of the entangled alginate structural network against applied forces (Wloka et al., 2005). While the importance of alginate acetyl groups for cell aggregation and microcolony formation *in vitro* is well established, the influence of *O*-acetyl groups on biofilm formation phenotypes in clinically relevant *P. aeruginosa* infections or related *in vivo* model systems of infection remain uncharacterized.

**Table 2:** Biological implications of EPS modifications.

Modification	Proteins involved	Organism studied	Implication of modification	Reference
**Alginate**
Acetylation	AlgF, AlgI, AlgJ, AlgX	*Pseudomonas aeruginosa*	Required for surface attachment and formation of structured microcolonies	Nivens et al. (2001), Tielen et al. (2005)
			Increased polymer viscosity	Tielen et al. (2005)
			Decreased neutrophil locomotion and lymphocyte transformation	Mai et al. (1993)
			Reduced activation of complement and opsonic killing by phagocytes	Pier et al. (2001)
			Scavenging of ROS	Learn et al. (1987)
			Reduced susceptibility to enzymatic degradation	Farrell and Tipton (2012)
			Increased gel thickness	Skjåk-Braek et al. (1989)
Epimerization	AlgG, AlgE1-7 (*Azotobacter vinelandii)*	*P. aeruginosa*	Improved gel forming ability (cohesion)	Grant et al. (1973), Donati et al. (2005)
			Upregulation of virulence factors through Ca^2+^ sequestration	Horsman et al. (2012)
		*A. vinelandii*	Maintain biofilm structure during changing environmental conditions	Ertesvåg et al. (1998)
			Preserve N-fixing capability	Sabra et al. (2000)
			Required for formation of functional cyst coat	Steigedal et al. (2008)
**PEL**
Deacetylation	PelA	*P. aeruginosa*	Required for biofilm formation (in PSL deficient strains)	Colvin et al. (2013)
**Cepacian**
Acetylation	BceOSU	*Bcc*	Reduced susceptibility to enzymatic degradation	Cescutti et al. (2006)
			Scavenging of ROS	Cuzzi et al. (2012)
***Vibrio* polysaccharide (VPS)**
Acetylation	VpsG	*Vibrio cholerae*	Required for robust biofilm formation and wild-type phenotypes	Fong et al. (2010)
**Poly-*N*-acetyl-glucosamine (PNAG)**
Deacetylation	PgaB (*Escherichia coli*), HmsF (*Yersinia pestis*) IcaB (*Staphylococcus epidermidis* and *S. aureus*)	*S. epidermidis*	Required for biofilm formation and surface attachment	Vuong et al. (2004a)
			Resistance to human cationic antimicrobial peptides	
			Resistance to neutrophil phagocytosis	
			Persistence in mouse model of infection	
		*S. aureus*	Required for biofilm formation and surface attachment	Cerca et al. (2007)
			Resistance to phagocytosis	
			Persistence in mouse model of infection	
		*E. coli*	Required for export of polymer and biofilm formation	Itoh et al. (2008)
		*Y. pestis*	Required for biofilm formation	Forman et al. (2006)
Succinylation	IcaC	*S. aureus*	Modulation improves *in vitro* fitness	Brooks and Jefferson (2014)

*P. aeruginosa* biofilm formation in the CF lung has been shown to provide significant protection from a variety of host immune factors. For example, decreased locomotion of neutrophils, as well as reduced lymphocyte transformation, have been observed when these cell types are incubated with alginate (Simpson et al., 1988). However, chemical removal of acetyl groups from alginate led to a complete loss of these inhibitory effects on neutrophil and lymphocyte function, suggesting that alginate *O*-acetylation is essential for their suppression (Mai et al., 1993). The activation of complement is also affected by the presence of acetyl groups (Pier et al., 2001). This is not surprising given that interactions between alginate and the complement component C3b likely occurs through unsubstituted hydroxyl groups (Hostetter et al., 1982), suggesting that the addition of acetyl groups to alginate in *P. aeruginosa* may have evolved as a mechanism for complement evasion. Activation of the alternative pathway of complement can lead to phagocytic killing, which is also impaired by the presence of *O*-acetyl groups. Opsonic killing of the FRD1 *O*-acetylation deficient mutant by phagocytes was readily observed, while wild-type FRD1 was resistant to these attacks (Pier et al., 2001). Alginate is also known to scavenge ROS produced by phagocytic cells during infection. Hypochlorite is a common ROS produced by phagocytes, and the presence of alginate in mucoid *P. aeruginosa* provides a significant protective advantage against hypochlorite over non-mucoid cells *in vitro* (Learn et al., 1987). This protective effect was, in part, attributed to the *O*-acetyl groups, as chemically deacetylated alginate exhibited impaired hypochlorite scavenging. Furthermore, addition of hypochlorite to native alginate led to a decrease in viscosity, similar to that seen for the chemically deacetylated alginate, suggesting that hypochlorite may be specifically reacting with *O*-acetyl groups from native alginate (Learn et al., 1987).

When bacteria are contending for control of the same environment, they can release extracellular enzymes to degrade critical structural components of cohabiting organisms to give them a competitive advantage (Korotkov et al., 2012). This is observed in the CF lung, where instances of multi-species biofilms are common (Elias and Banin, 2012). During colonization of the CF lung, alginate acetyl groups may serve as a protective mechanism to prevent unwanted degradation of alginate within the biofilm by bacteria that could secrete an AlgL-like lyase as an offensive tactic. The *P. aeruginosa* alginate lyase AlgL preferentially degrades deacetylated alginate or polyM over mature, acetylated alginate (Farrell and Tipton, 2012). Furthermore, *O*-acetyl groups prevent the epimerization of ManA to GulA by the epimerases AlgE1-7 in *A. vinelandii*, which may allow for control over the degree of epimerization and, in turn, regulation of the cyst coat composition.

Alginate acetylation content ranges from 4 to 57%, depending on the percentage of ManA present (Skjåk-Braek et al., 1986). The degree of *O*-acetylation is often observed to vary not only between different alginate-producing organisms, but also between different strains of the same organism and even within the same strain under differing growth conditions (Marty et al., 1992; Peña et al., 2006). For example, modulation of carbon source during growth for a single alginate-producing *P. syringae* strain led to significant differences in acetyl content, ranging from 9 to 34% of total uronic acids bearing an acetyl group (Day, 1988). In another study, the alginate produced by several different strains of *P. aeruginosa* grown on nutritionally distinct media was examined. This study revealed that between different strains *O*-acetyl content of alginate varied between 2 to 56% (Marty et al., 1992). Furthermore, in both studies alginate acetyl content changed over the course of a single growth experiment by as much as 40%, possibly owing to the availability of acetyl-CoA, the proposed acetyl donor (Lee and Day, 1998). In addition to acetyl-CoA availability, differences in acetyl content could conceivably be a means to optimize attachment, nutrient uptake, or nutrient diffusion within the biofilm in the face of different media compositions and nutrient sources. This notion is supported by findings which suggest that alginate *O*-acetylation can enhance the swelling ability of calcium alginate gels (Skjåk-Braek et al., 1989). Deacetylated alginate exhibited poor swelling ability in comparison to chemically acetylated variants, with increasing degrees of acetylation leading to greater swelling volume. Conversely, increased *O*-acetylation led to a decrease in the affinity of alginate gels for calcium ions (Skjåk-Braek et al., 1989). Thus, alginate acetyl content has specific consequences with respect to calcium ion binding and the thickness of alginate gels, which may influence nutrient diffusion in the biofilm. These findings could potentially be extrapolated to other components of the growth media, and suggests a mechanism by which alginate-producing bacteria could regulate the uptake of essential nutrients.

## Alginate Epimerization

*Pseudomonas aeruginosa* has a single alginate C5-epimerase in the periplasm, AlgG. In *A. vinelandii*, there is an AlgG ortholog that performs the same function, and seven additional extracellular epimerases, AlgE1 through AlgE7 (**Table 1**). Alginate can form strong gels through interactions with GulA residues, mediated by Ca^2+^ ions. This feature was thought to be limited to alginates containing G-blocks, and would therefore exclude the MG-block alginates produced by *P. aeruginosa* (Grant et al., 1973). However, it is now thought that alginates containing exclusively MG-blocks can also form gels in the presence of Ca^2+^ (Donati et al., 2005), suggesting that epimerization by AlgG in *P. aeruginosa* may serve as a mechanism to improve the cohesion of alginate during biofilm formation. It was found that addition of CaCl_2_ to growth media led to the production of biofilms that were 10- to 20-fold thicker than that produced in the absence of Ca^2+^ (Sarkisova et al., 2005). Ca^2+^-alginate interactions also regulate virulence factor expression, as chelation of Ca^2+^ by alginate induces expression of the Type 3 secretion system (Horsman et al., 2012). Therefore, it appears that there are mechanisms in place in *P. aeruginosa* for virulence factors to be upregulated by the expression of another, thus allowing for concerted actions that improve fitness (**Table 2**). Despite advances in understanding the interplay between Ca^2+^ and alginate in *P. aeruginosa*, and the extensive studies performed on acetyl-deficient alginate, there are no reports on the effects of epimerization on biofilm formation, pathogenicity, or virulence.

In contrast, the role of epimerization in *A. vinelandii* cyst formation has been well characterized. One hypothesis regarding the ability of *A. vinelandii* to express multiple epimerases with unique activities is that these enzymes allow the alginate structures to be tailored to different layers of the cyst under diverse environmental conditions. For example, the epimerase AlgE1 has two catalytic domains that introduce primarily MG-blocks and G-blocks, respectively (Ertesvåg et al., 1998). Decreasing the availability of Ca^2+^ in the presence of AlgE1 *in vitro* led to greater incorporation of G-blocks into polyM alginate. This may provide a means *in vivo* to maintain the strength of Ca^2+^-mediated inter-alginate bonds in the face of decreased environmental Ca^2+^ availability (Ertesvåg et al., 1998). Regulation of alginate structure is also observed during vegetative growth of *A. vinelandii*, where nitrogen fixation is mediated by the expression of highly oxygen-sensitive nitrogenases. In this state, alginate is utilized as a barrier to prevent the diffusion of oxygen into the cell. In the presence of increasing environmental O_2_ concentrations, *A. vinelandii* was able to produce alginate with greater G-content, which led to the formation of a thicker, denser alginate layer around the cell and thus limited oxygen penetration (Sabra et al., 2000). The expression of different mannuronan C5-epimerases is also regulated over the course of the *A. vinelandii* life cycle, including during vegetative growth, cyst development, and cyst germination (Høidal et al., 2000). Although the exact biological function for the expression of specific epimerases at unique points in the life cycle of *A. vinelandii* has not been determined, preferential expression of AlgE7 during cyst germination could be linked to the apparent lyase activity of this enzyme, which may be utilized to degrade the cyst coat (Høidal et al., 2000).

Unlike AlgG in *P. aeruginosa*, the importance of the AlgE1-7 epimerases in the formation of the cyst coat and tolerance to desiccation has been explored. Inactivation of the AlgE1-7 epimerases, either through chromosomal deletion in *A. vinelandii* (Steigedal et al., 2008) or by inactivating the Type 1 secretion system responsible for their export (Gimmestad et al., 2006) led to the production of low G-content alginate, suggesting that the periplasmic *A. vinelandii* AlgG is active but not very efficient. In both cases, these mutants were unable to form a cyst coat and could not survive desiccation. In contrast, deletion of individual *algE* genes, with the exception of *algE3*, did not have an appreciable effect on the G-content of alginate. Deletion of *algE3* showed a significant reduction in G-content (Steigedal et al., 2008). However, since each of the individual *algE* deletion mutants was able to form a functional cyst and survive desiccation, it appears that no single epimerase is absolutely essential for cyst formation or germination. This suggests that the presence of multiple extracellular epimerases may increase redundancy of epimerase activity to ensure formation of a functional cyst coat (Steigedal et al., 2008). It remains to be determined whether cyst formation under unique stressful conditions may require specific epimerases, and little work has been done to date to examine the role of mannuronan C5-epimerases during the vegetative stage of *A. vinelandii* growth.

While a great deal is understood about the regulation of alginate biosynthesis and its modification at the genetic and protein level (Hay et al., 2014), the implications of alginate acetylation and epimerization in terms of biofilm formation, pathogenicity and virulence, environmental adaptability and survivability remain largely uncharacterized.

## The Pel Polysaccharide

In addition to alginate, *P. aeruginosa* is capable of synthesizing two other polymers that have been implicated in biofilm formation, the Pel and Psl polysaccharides (PEL and PSL; Franklin et al., 2011). Unlike alginate, PEL and PSL are primarily associated with the establishment of non-mucoid biofilms. PSL is a neutral, branched polysaccharide with a five-sugar repeat unit composed of D-mannose, D-glucose, and L-rhamnose and is not thought to undergo any modifications after polymerization (Byrd et al., 2009). The exact structure of PEL is currently unknown, but it is predicted to be glucose rich (Friedman and Kolter, 2004; Ma et al., 2012). Colvin and colleagues have demonstrated that the PEL biosynthesis protein PelA has deacetylase activity *in vitro* and when residues predicted to be required for deacetylation were mutated, this activity was lost. Introduction of these PelA deacetylation mutations in *P. aeruginosa* PA14, which uses PEL as the primary EPS, led to a biofilm deficient phenotype and a lack of material recognizable by PEL-reactive antisera (**Table 1**; Colvin et al., 2013). Given the localization of PelA to the periplasm (Colvin et al., 2013), deacetylation of PEL following polymerization may be necessary for biofilm formation, and suggests that an acetylated sugar is likely a feature of the PEL. Our current understanding of PEL biosynthesis is limited, and it remains to be determined whether PelA acts directly on PEL, the degree of PEL deacetylation by PelA, and what effect this modification has on virulence.

## Cepacian

**FIGURE 4:**
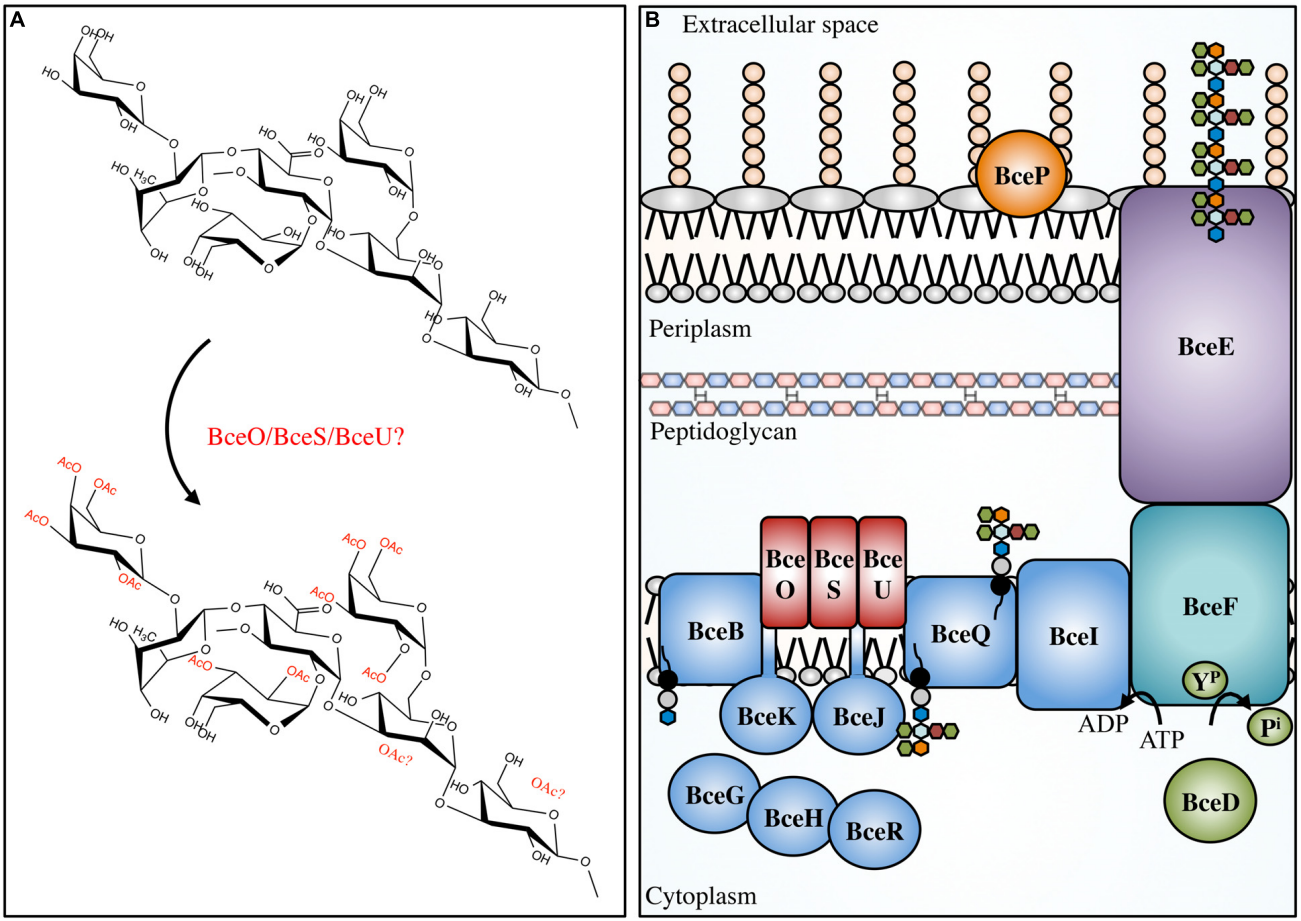
**Cepacian modifications and biosynthetic apparatus.** *Not to scale.* **(A)** Cepacian may be *O*-acetylated at various locations by BceOSU, leading to a number of unique combinations with an average of three acetyls per repeat unit. **(B)** Cepacian repeat units are synthesized in the cytoplasm on an isoprenoid lipid carrier (black circle/gray circle), initiated by BceB and continued by glycosyltransferases BceGHJKR. BceGHR are cytoplasmic, while BceJK are integral membrane proteins. BceOSU are predicted to be membrane embedded acetyltransferases that decorate the repeat unit with acetyl groups. BceQ translocates the repeat units across the inner membrane, followed by addition of repeat units to the growing polymer at the periplasmic side, which is dependent on BceI. Polymerization and export requires BceF, a tyrosine kinase. BceD is a protein tyrosine phosphatase which dephosphorylates BceF. BceE is the channel for polymer export across the OM. BceP is putatively involved in polysaccharide degradation, though its role and localization is unkonwn. ATP, adenosine-5′-triphosphate; ADP, adenosine-5′-diphosphate; Y^P^, phosphorylated tyrosine residue; P^i^, inorganic phosphate. Green hexagon, galactose; orange hexagon, GlcA; light blue hexagon, mannose; maroon hexagon, rhamnose; blue hexagon, glucose.

The Bcc is a group of at least 17 different bacterial species, including beneficial environmental isolates, as well as rhizosphere parasites, and plant and animal pathogens (Mahenthiralingam et al., 2002; Vanlaere et al., 2009). The Bcc have become increasingly important as opportunistic pathogens of immunocompromised individuals and those with CF (Mahenthiralingam et al., 2005). In CF patients, Bcc infections occasionally develop into a form of necrotising pneumonia, known as cepacia syndrome, which often leads to patient death (Govan and Deretic, 1996). The majority of both clinical and environmental Bcc isolates produce the EPS cepacian (Ferreira et al., 2010); a known virulence factor that contributes significantly to bacterial pathogenicity. Cepacian is composed of glucose, GlcA, mannose, rhamnose, and galactose in a 1:1:1:1:3 ratio and is decorated with acetyl groups (**Figure 4**; Cérantola et al., 1999; Cescutti et al., 2000; Linker et al., 2001). The acetyl groups can be found at 12 different locations on the polymer repeating unit with an average of three acetyl groups present per repeat unit (Cescutti et al., 2011). The genes responsible for cepacian acetylation were discovered by Ferreira and colleagues, and include the putative acetyltransferases *bceO*, *bceS*, and *bceU* (Ferreira et al., 2010). Mutations in *bceS* produced cepacian with approximately 20% fewer acetyl groups, implicating this protein in partial cepacian acetylation (Ferreira et al., 2010). The roles of *bceO* and *bceU* in cepacian acetylation have not been evaluated, nor has a triple mutant been generated to discern the fitness of an acetyl-deficient cepacian producer.

An enzyme with lyase activity that specifically degrades cepacian has been isolated from culture supernatants of *Bacillus* sp. This enzyme has significantly higher activity against the chemically deacetylated polymer than native cepacian, implicating acetylation in protective mechanisms (**Table 2**; Cescutti et al., 2006). As with alginate acetylation, cepacian acetylation may have evolved as a defensive mechanism to prevent polymer cleavage in the presence of competitive organisms. Furthermore, like alginate, cepacian acetyl groups have been shown to provide protection against ROS, specifically hypochlorite (Cuzzi et al., 2012). Acetyl groups were the first structural features to undergo damage following hypochlorite treatment, which led to a loss of polymer-polymer interactions and cepacian unfolding, increasing the susceptibility of the cepacian backbone to hypochlorite degradation (Cuzzi et al., 2012). Consequently, cepacian acetylation improves polymer robustness to hypochlorite-mediated damage and increases the amount of polymer reactive groups that could neutralize hypochlorite prior to reaching the cellular surface. Further study of cepacian producing Bcc pathogens is necessary to determine, whether like alginate, there is a role for cepacian acetyl modification in immune evasion.

## *Vibrio* Polysaccharide (VPS)

*Vibrio cholerae* is a human pathogen that causes the diarrhoeal disease cholera (Kaper et al., 1995; Faruque et al., 1998). This bacterium is a natural inhabitant of aquatic ecosystems, where it forms biofilms on a variety of surfaces, including plankton, plants, crustaceans, insects, and sediment (Huq et al., 1983, 1995; Halpern et al., 2004; Broza et al., 2005). In areas where cholera is endemic, *V. cholerae* has been shown to form suspended biofilm-like aggregates in surface waters, however, when particles >20 μm in diameter are removed from water sources, the incidence of cholera can be reduced (Huq et al., 1996; Colwell et al., 2003). Furthermore, it has been shown that the average infectivity of the aggregate form of *V. cholerae* is significantly higher than that of planktonic cells (Faruque et al., 2006), and biofilm formation within aquatic ecosystems significantly improves *V. cholerae* fitness and persistence (Matz et al., 2005). A major component of the biofilm produced by *V. cholerae* is an EPS called VPS. This polymer is thought to be produced during infection and contributes to bacterial colonization and survival (Yildiz and Schoolnik, 1999; Fong et al., 2010). The chemical structure of VPS revealed a backbone containing the unusual constituent GulNAcAGly: the amide formed from 2-acetamido-2-deoxy-L-guluronic acid and glycine (**Figure 6**; Yildiz et al., 2014). Of the genes involved in VPS biosynthesis, originally identified using a transposon mutagenesis screen (Yildiz and Schoolnik, 1999), two putative acetyltransferases, *vpsG* and *vpsC*, were identified (Fong et al., 2010). Deletion of *vpsG* results in reduced biofilm formation and altered biofilm-related phenotypes, as well as weak reactivity with VPS antisera, suggesting that it may modify the polymer, perhaps through acetylation (**Table 1**). In contrast, deletions of *vpsC* do not affect biofilm formation or VPS production, suggesting that *vpsC* is inactive, not expressed, or is performing some other function in VPS biosynthesis besides polymer modification (Fong et al., 2010). The chemical composition of VPS produced by *vpsG* and *vpsC* mutants was not studied for alterations in acetyl content. The presence of GulNAcA in VPS may be the result of epimerization by the predicted GDP-mannose dehydrogenase VpsB, a conversion similar to the ManA to GulA epimerization catalyzed by AlgG in the biosynthesis of alginate by *P. aeruginosa* (Wolfram et al., 2014; Yildiz et al., 2014). The unusual glycine modification in VPS requires further exploration, as the enzyme responsible for its addition is presently unknown. Given the important role of VPS in *V. cholerae* pathogenesis and environmental persistence, and the recent determination of its precise chemical structure, we anticipate that the proteins involved in VPS modification will soon be identified and characterized.

## Glycosaminoglycans (GAGs)

GAGs are a group of polymers that are typically composed of a disaccharide repeat unit containing an amino sugar and a hexuronic acid (Laurent and Fraser, 1992; Esko and Lindahl, 2001; DeAngelis, 2002; Silbert and Sugumaran, 2002). GAGs were initially thought to exist only in the animal kingdom, where they serve essential biological functions, however, there has been an emergence of GAG-like polymers amongst prokaryotes (Vann et al., 1981; Rodriguez et al., 1988; DeAngelis et al., 2002). Prokaryotic GAGs are typically less complex than their eukaryotic counterparts due to an absence of modifications such as sulfation (Raedts et al., 2011). HS, for example, is an essential GAG in animals and is composed of repeating disaccharides of GlcA and GlcNAc (Kjellén and Lindahl, 1991). HS can be modified post-polymerization by a glucuronyl C5-epimerase, which converts GlcA to IdoA, as well as by the addition of sulfate groups to GlcNAc or IdoA moieties. Mouse embryos lacking the GlcA C5-epimerase display a lethal phenotype characterized by skeletal malformations and lung defects (Li et al., 2003), highlighting the importance of HS epimerization in murine development. Interestingly, the K5 antigen of *E. coli* O10:K5:H4 has an identical structure to heparosan, the unsulfated, non-epimerized backbone structure of HS (Vann et al., 1981). K5 heparosan is a form of molecular camouflage, as it imparts low immunogenicity to the bacterium in humans and hence increased pathogenicity (Vann et al., 1981).

Although sulfation has not yet been observed amongst prokaryotic GAGs, IdoA residues have been found to be constituents of bacterial GAGs (**Figure 6**). The identification of the bacteria glucuronyl C5-epimerase has proven elusive (Raedts et al., 2011), however, an enzyme (RED65_08024) from the marine bacterium *Bermanella marisrubi* that shares 37% sequence similarity with the human glucuronyl C5-epimerase has been characterized and shown *in vitro* to convert GlcA to IdoA in de-sulfated mouse HS (Raedts et al., 2013). This glucuronyl C5-epimerase represents the first prokaryotic protein capable of generating IdoA residues, and is the only identified epimerase that can function on bacterial polysaccharides post-polymerization, besides AlgG and AlgE1-7. Unfortunately, the EPS produced by *B. marisrubi* has not been characterized, so its target remains unknown. Nevertheless, the ability of bacteria to more closely replicate the structures of essential human polysaccharides by expression of homologous modification enzymes likely serves as a mechanism to mask their presence from the host immune system (Cress et al., 2014).

## Poly-β-1,6-*N*-Acetyl-glucosamine (PNAG)

Poly-β-1,6-*N*-acetyl-glucosamine is a poly-GlcNAc polymer that is produced by a wide range of Gram-positive and Gram-negative bacterial pathogens, including *Staphylococcus epidermidis*, *Staphylococcus aureus*, *Escherichia coli*, *Yersinia pestis*, *Bordetella* sp., *Acinetobacter baumanii*, *Actinobacillus pleuropneumoniae*, *Burkholderia cepacia* complex (Bcc), and *Aggregatibacter actinomycetemcomitans* (Cramton et al., 1999; Vuong et al., 2004b; Wang et al., 2004; Izano et al., 2007, 2008; Parise et al., 2007; Bobrov et al., 2008; Choi et al., 2009). These organisms are responsible for a wide spectrum of diseases, including but not limited to, hospital acquired infections, toxic shock syndrome, plague, and whooping cough. Depending on the source or organism in question, PNAG may also be referred to as PGA (polyglucosamine, in Gram-negative bacteria), PIA (in Gram-positive bacteria), poly-NAG, hms+ (in *Y. pestis*), or BPS (*Bordetella* polysaccharide, in *Bordetella* sp.). Given the differences in PNAG modifications between Gram-positive and Gram-negative bacteria, as described below, we will use PGA and PIA to refer to PNAG polymer produced by Gram-negative and Gram-positive organisms, respectively.

**FIGURE 5:**
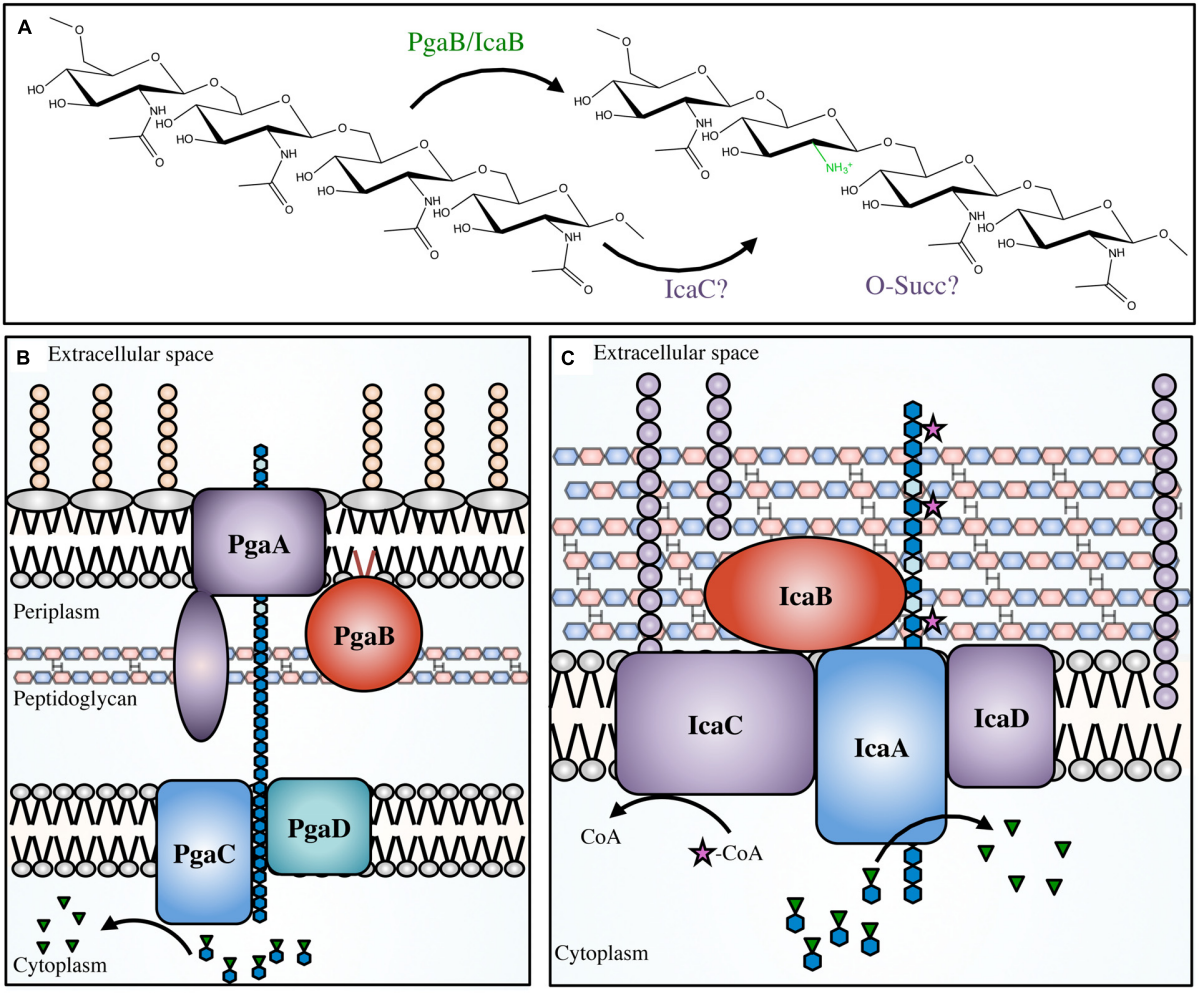
**The PNAG and PIA modifications and biosynthetic apparatus.** *Not to scale.* **(A)** PNAG polymers are partially deacetylated by PgaB in *Escherichia coli*, or IcaB in *Staphylococcal* species. It has been proposed that IcaC *O*-succinylates the polymer in certain *Staphylococcal* species, however, the location of the succinyl groups and the order of deacetylation/succinylation has not yet been determined. The proteins involved in PNAG biosynthesis in *E. coli* **(B)**, and PIA synthesis in *Staphylococcal* species **(C)**. PNAG (blue hexagon chain) is synthesized in the cytoplasm from the nucleotide-sugar precursor UDP-GlcNAc (blue hexagons with green inverted triangles). The polymer is transported across the inner membrane *via* PgaCD, deacetylated (light blue hexagons) by PgaB in the periplasm, and then exported through the PgaA porin. PIA is transported across the cytoplasmic membrane by IcaAD, then partially deacetylated by IcaB in the extracellular space. PIA has been proposed to be *O*-succinylated by IcaC (magenta star).

Initially *S. epidermidis* was thought to produce several different polymers, but the discovery of the *icaADBC* operon (Heilmann et al., 1996a,b; Gerke et al., 1998) revealed that only a single polymer, PIA, was produced (Tojo et al., 1988; Christensen et al., 1990; Heilmann et al., 1996a; Mack et al., 1996; McKenney et al., 1998). PIA is a partially deacetylated β-1,6-GlcNAc polymer. In *S. epidermidis* and *S. aureus* 15–20% of the *N*-acetyls are removed by the extracellular, cell-surface associated polysaccharide deacetylase IcaB (**Table 1**; **Figure 5**; Vuong et al., 2004a; Cerca et al., 2007). In addition to deacetylation, approximately 6 and 10% of GlcNAc residues in *S epidermidis* and *S. aureus*, respectively, are *O*-succinylated (Joyce et al., 2003; Sadovskaya et al., 2005). This modification is thought to be performed by the membrane localized protein, IcaC (Atkin et al., 2014). Interestingly, a mechanism of phase variation, where bacteria modulate virulence phenotypes at the genome level in a rapid on/off fashion, was noted in *S. aureus* wherein slipped-strand mispairing led to inactivation of *icaC* (Brooks and Jefferson, 2014). This phenotype confers a fitness advantage that was not seen when the *ica* operon was deleted, which may be a response to modulate PIA *O*-succinylation and thus decrease the overall anionic charge of the polymer.

**FIGURE 6:**
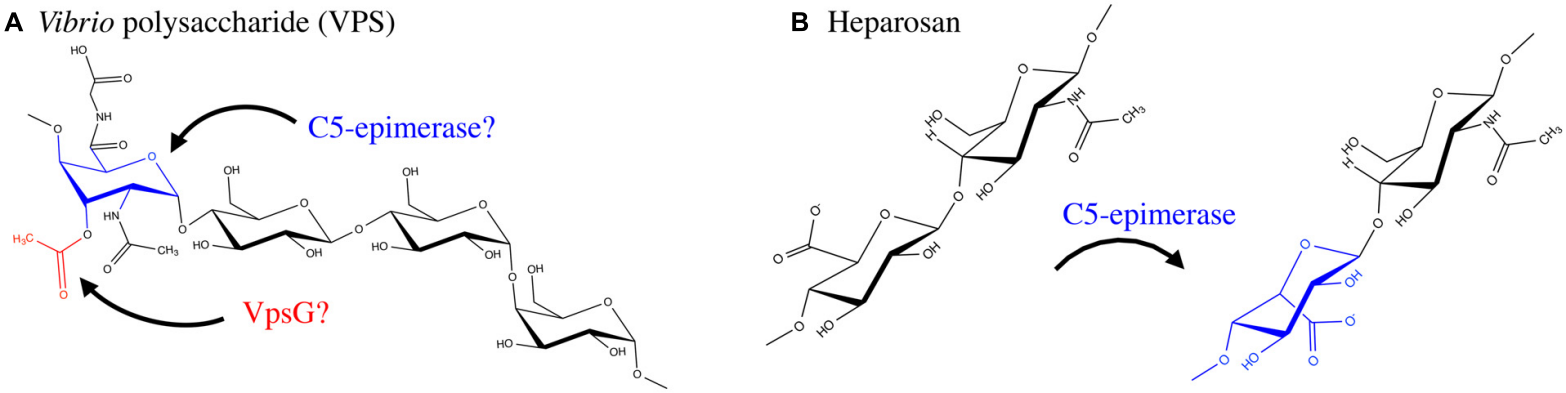
**Exopolysaccharide modifications of VPS and Heparosan. (A)** VPS is produced by *Vibrio cholerae* O1 El Tor and contains an *O*-acetyl group, likely added by VpsG. In addition the polymer has a glycine modification and an *N*-acetyl group; the enzymes responsible for these modifications have not been determined. VPS contains a GulA residue which is epimerized by an unknown C5-epimerase. **(B)** Heparosan, a GAG composed of a disaccharide repeating unit of GlcA and GlcNAc, produced by select bacteria such as the urinary tract pathogen *E. coli* O10:K5:H4. A C5-epimerase introduces IdoA residues. The proteins involved in, and mechanism of, biosynthesis of the above EPS have not been fully resolved.

The production of PGA has been extensively characterized in *E. coli*, where the *pgaABCD* operon encodes the proteins necessary for its biosynthesis (Wang et al., 2004). In *E. coli*, approximately 3–5% of *N*-acetyls are removed by the lipoprotein PgaB (Wang et al., 2004; Itoh et al., 2008; Little et al., 2012). The N-terminal domain of PgaB is homologous to IcaB in Gram-positive bacteria but the protein is located on the inner leaflet of the outer membrane. There is no IcaC ortholog in the *pgaABCD* operon, which is consistent with an observed lack of *O*-succinyl groups in PGA.

Partial deacetylation of PNAG by both IcaB and PgaB is important for a variety of biofilm-associated phenotypes in *S. epidermidis*, *S. aureus*, and *E. coli*. Deletion of *icaB* in *S. epidermidis* led to the production of fully acetylated PIA, suggesting that IcaB is not necessary for polymer production (Vuong et al., 2004a). However, the fully acetylated polymer was not retained at the cellular surface and was shed into the culture media, which led to deficiencies in biofilm formation and surface attachment (**Table 2**). The lack of deacetylation led to a loss of cationic charge in the polymer, which may be essential for interactions with the anionic cell surface of *S. epidermidis* (Vuong et al., 2004a). Furthermore, *icaB*-deficient mutants of *S. epidermidis* were more susceptible to human cationic antimicrobial peptides and phagocytosis by neutrophils, and were unable to persist in a mouse model of device-related infections (Vuong et al., 2004a). Deletion of *icaB* in *S. aureus* produced similar phenotypes (Cerca et al., 2007). Intriguingly, the production of wall teichoic acids, the predominant anionic component of the Gram-positive bacterial envelope, was dispensable for adherence of PIA to the cell surface of *S. aureus* (Vergara-Irigaray et al., 2008), suggesting that other less prevalent anionic species mediate this interaction.

In contrast to IcaB, inactivation of PgaB in *E. coli* prevented polymer export, suggesting that partial deacetylation is necessary for export through the predicted outer membrane porin PgaA (Itoh et al., 2008). This is in line with findings that suggest conformational changes in the C-terminal domain of PgaB, upon binding of deacetylated PNAG, assist in targeting PNAG for export (Little et al., 2014b). Deacetylation has also been studied in *Y. pestis*, where a PNAG-like polymer is thought to mediate biofilm formation. Biofilm formation in *Y. pestis* is crucial for its zoonotic transmission (Jarrett et al., 2004). In the flea, the proventriculus, a feeding tube covered in spines that connects the midgut to the esophagus, provides a platform for the adhesion of *Y. pestis* aggregates. Subsequent colonization impedes blood passage and leads to transposition of *Y. pestis* from flea to mammal when a flea attempts excessive feeding due to a partial or completely blocked proventriculus (Jarrett et al., 2004). The *hmsHFRS* operon in *Y. pestis* is orthologous to the *pgaABCD* operon, where HmsF is the outer membrane localized deacetylase with structural similarity to PgaB (Forman et al., 2006). Mutation or deletion of *hmsF* led to a deficiency in biofilm formation. This suggests HmsF in *Y. pestis* may be analogous to PgaB in *E. coli* in terms of de-*N*-acetylation activity and importance for polysaccharide export and biofilm formation.

While PNAG production has been studied primarily in *S. aureus*, *S. epidermidis*, and *E. coli*, there are a multitude of additional pathogenic bacteria, fungi, and protozoans that may produce this polymer (Cywes-Bentley et al., 2013). PNAG could represent the first example of an EPS that is broadly utilized by pathogenic organisms as a mechanism to improve fitness in the environment or during infection.

## Insights from Modification of Other Microbial Polysaccharides

The implications of EPS modifications in pathogenic bacteria have been studied to some extent, particularly in alginate and PNAG producing bacteria (**Figure 2**; **Table 2**). However, the breadth of our knowledge in this field remains limited. Despite this, comparable modifications found on LPS and CPSs have been studied extensively in an effort to identify vaccine targets, and can be used for comparison purposes to generate new hypotheses regarding EPS modifications (Cody et al., 2003; Pinto and Berti, 2014). In particular, the study of polysaccharide acetyl modifications has clarified their role in mediating a variety of survival mechanisms.

Many of the protective benefits of EPS acetyl modifications described above have been noted for other bacterial pathogens. For instance, in *Haemophilus influenzae*, an opportunistic pathogen of the upper respiratory tract, acetylation of LPS by the acetyltransferase OafA leads to increased resistance to complement-mediated killing by human serum (Fox et al., 2005). Similarly, in *S. aureus*, the acetyltransferase Cap5H, which is responsible for the *O*-acetylation of type 5 CPS, confers protection against opsonophagocytic killing and improves propagation in a murine model of infection (Bhasin et al., 1998). Type 5 CPS producers also exhibit increased survival rates in murine models of bacteremia and renal abscess formation and resistance to killing in whole mouse blood and opsonophagocytic assays, in comparison to producers of the structurally similar type 8 CPS which have reduced levels of *N*-acetylation (Watts et al., 2005). Beyond the prokaryotic domain, acetyl modifications are also incorporated into the CPS of the pathogenic fungus *Cryptococcus neoformans* to evade complement activation (Fujihara et al., 1997), decrease the efficiency of capsule clearance by the host (Kozel et al., 2003), and inhibit neutrophil migration (Ellerbroek et al., 2004) during cryptococcosis. An excellent example of the benefits of polysaccharide acetyl modifications comes from a survey of clinical isolates of *Streptococcus pneumoniae* and *E. coli* K1, which found that the bacteria expressing acetyl-decorated polymers were more virulent and invasive than those that expressed polymers lacking the modification (Frasa et al., 1993; Melin et al., 2010). Therefore, the immunomodulatory characteristics of acetyl modifications are utilized by a wide range of pathogenic organisms and likely represent a general mechanism for survival and proliferation within the host.

The above notion is firmly supported by studies of serotype variation within the context of CPS biosynthesis. In *Streptococcus pneumoniae*, a causative agent of meningitis, bacteremia, and pneumonia, there are more than 90 different capsule serotypes with unique carbohydrate structures and biosynthetic loci. This has evolved, in part, as a mechanism to overcome serotype-specific host mechanisms of adaptive immunity that can efficiently clear infections. In some serotypes, such as 9V/9A, 11A/11E, and 15B/15C, the CPS structures differ only in the degree of *O*-acetylation (Jansson et al., 1987; Rutherford et al., 1991; Zartler et al., 2009). Mechanisms within *S. pneumoniae* have been revealed that allow for serotype switching during infection as a means to actively evade the host immune response. In the case of serotypes 9V and 11A, inactivating mutations in the acetyltransferase-encoding gene *wceJ* led to expression of non-acetylated 9A and 11E capsule (Calix and Nahm, 2010; Calix et al., 2011). Moreover, certain *wceJ* mutations only partially inhibit acetyltransferase activity, which have led to intermediate 9V/9A and 11A/11E phenotypes (Calix et al., 2011, 2014). In the case of 15B/15C serotype switching, the process is reversible due to slipped-strand mispairing of the acetyltransferase-encoding gene *wciZ* (van Selm et al., 2003). Regardless of the mechanism, this mid-infection serotype variation provides significant protective advantages to *S. pneumoniae* in terms of antibody evasion. For example, antibodies generated against *O*-acetylated serotype 15B were unreactive with non-acetylated 15C polymer (Rajam et al., 2007), and serotype 9V specific antibodies exhibited reduced specificity for 9A polymer (Calix et al., 2012). Furthermore, 10–20% of individuals receiving a *S. pneumoniae* vaccine targeted against the 9V polysaccharide did not generate antibodies targeting serotype 9A (McNeely et al., 1998). In addition, the ability of acetyl groups to mask protective epitopes of bacterial polysaccharides has been noted for the Vi antigen of *Salmonella typhi* (Szu et al., 1991), *Salmonella typhimurium O*-antigen (Kim and Slauch, 1999) and *Neisseria meningitidis* serogroup A, C, and Y CPS (Michon et al., 2000; Berry et al., 2002; Fusco et al., 2007). Therefore, through modulation of acetyl groups on the polymer, a wide variety of pathogenic bacteria are able to evade host-mediated mechanisms of adaptive immunity.

The above examples illustrate scenarios in which acetyl modification is an all-or-nothing response to adaptive immunity, however, in the case of GBS, acetyl levels on its sialic acid CPS can be fine-tuned by the actions of the acetyltransferase NeuD and the acetylesterase NeuA (Lewis et al., 2006, 2007). Different degrees of *O*-acetylation in GBS CPS have been linked to different stages of invasion and infection. For instance, it is thought that during the asymptomatic stages of initial colonization and persistence in the human gastrointestinal and vaginal tracts, GBS produces an extensively acetylated form of CPS to protect against degradation by sialidases introduced by competing microbes in these environments (Weiman et al., 2009). However, highly acetylated CPS renders GBS more susceptible to killing by neutrophils and reduces virulence during stages of opportunistic infections (Weiman et al., 2010). Therefore, during infection it is thought that GBS produces a sparsely acetylated form of CPS that improves resistance to neutrophil-mediated killing through reduced neutrophil activation and production of pro-inflammatory cytokines, and enhances survival in the murine bladder (Kline et al., 2011). Interestingly, this variant of CPS is also able to promote the persistence of uropathogenic *E. coli* in co-culture urinary tract infection models (Kline et al., 2012). Therefore, in certain pathogens, specific degrees of polysaccharide acetylation allow for adaptation during different stages of colonization and infection.

The above examples of acetyl modulation in LPS and CPS not only reinforce the importance of EPS acetylation for pathogenicity and persistence, but also provide additional perspectives in considering the variability of this modification observed in alginate and cepacian. For instance, cepacian has on average three acetyl groups per repeat unit, each located on one of 12 potential positions (Cescutti et al., 2011). Therefore, each cepacian repeat unit can have one or more acetyl groups at any of 12 positions, generating an overwhelming number of unique acetyl decoration patterns. Given the importance of acetyl groups in forming or masking antibody epitopes, this level of diversity would make the generation of protective antibodies or the development of an effective vaccine extraordinarily difficult. Furthermore, production of such a heterogeneous polymer likely requires an arsenal of regulatory factors and/or acetyltransferases, very few of which have been discovered in the context of cepacian biosynthesis (Ferreira et al., 2011). Similar to *S. pneumoniae* and *N. meningitidis* CPS production, members of the Bcc may modulate the presentation of cepacian acetyl groups through an as yet unknown mechanism as a means to evade host adaptive immune mechanisms.

The degree of acetylation and epimerization of alginate has long been known to differ depending on the organism and strain, as well as the composition of the growth medium (Day, 1988; Marty et al., 1992; Peña et al., 2006). This reflects, in part, a need to adapt to the specific conditions imposed by different nutritional media, and may mirror other features of the environment from which the organism was isolated. In line with this concept, additional promoters within the alginate operon have been identified upstream of *algG* and *algIJF* in *P. aeruginosa*, suggesting that there may be modes of regulating the levels of these modifying enzymes independently of the rest of the alginate biosynthesis machinery (Paletta and Ohman, 2012). The upregulation of *O*-acetylation machinery would not only increase alginate acetyl content, but would also decrease the availability of substrate for AlgG and thus decrease epimerization levels. Conversely, upregulation of *algG* would increase the number of G-residues that cannot act as substrates for *O*-acetylation. Therefore, there is the potential for a complicated regulatory interplay between these processes, much like the reciprocal *O*-acetylation/de-*O*-acetylation of GBS CPS that allows for fine-tuning of acetyl levels at different stages of infection.

The degree of PNAG de-*N*-acetylation is not known to vary considerably, and the exact processes involved in VPS acetylation and epimerization, PIA *O*-succinylation, PEL deacetylation and GAG epimerization are poorly understood. However, the ability to perform these types of modifications in a random fashion may increase the difficulty in generating antibodies that recognize specific epitopes on these EPS, either during host adaptive immune responses or in vaccine development (Gening et al., 2010). As such, EPS modifications are capable of imparting beneficial characteristics upon polymers that improve persistence, survival, or evasion of the immune response in their cognate bacteria regardless of their frequency, mechanism of addition to the polymer, or chemical properties.

## Reflection and Future Perspectives

Identifying and characterizing biofilm EPS is difficult and there are a number of hurdles that need to be overcome. One of the initial challenges involves culturing biofilm-forming bacteria. Identification of an appropriate medium and growth conditions is required to study EPS production of certain microorganisms in the laboratory (Stewart, 2012). Of those that can be cultured, it is imperative to use similar growth conditions when making experimental comparisons in the literature, as variations can affect the presence or degree of different polysaccharide modifications. With alginate, varying levels of acetylation and epimerization are observed depending on the culture conditions, as well as varying biofilm phenotypes of identical *P. aeruginosa* strains (Pier et al., 2001; Tielen et al., 2005). Additionally, conflicting studies on the levels of pyruvyl and *O*-acetyl modifications to xanthan gum were attributed to different media conditions (Bradshaw et al., 1983). Different media or culturing equipment may also affect experiments such as surface attachment assays. For example, different types of plastics were found to affect PIA-mediated surface attachment in microtitre plates (Maira-Litràn et al., 2004). This suggests that during preliminary analyses, multiple types of media and different materials including plastics and glass should be tested to ensure the validity of observed biofilm phenotypes. Variations in the abundance or type of modifications on a given polymer under different experimental conditions can be difficult to quantify; however, this variation likely reflects the ability of different bacteria to adapt to unique situations. Many EPS-producing bacteria naturally exist in diverse environments and are also able to infect various hosts and survive in specific tissues. Additionally, during the course of infection, the environment within the host will change as immune mechanisms attempt to eradicate the bacteria and the surrounding tissue suffers damage. Variations in the degree of modifications under different growth conditions or stressors may therefore provide valuable insight into bacterial adaptation.

The majority of biofilm studies focusing on EPS modifications have been performed using *in vitro* model systems with mono-species cultures. However, the majority of biofilms from chronic infections differ significantly from those studied in the laboratory (Bjarnsholt et al., 2013a). One significant issue lies in the use of abiotic surfaces, such as cover slides or the plastic surfaces of 96-well plates for the growth and study of biofilms. While some systems can closely approximate the conditions encountered *in vivo*, such as flow cell models for catheter associated infections, biofilms from infections like CF pneumonia or those encountered in epidermal wounds are thought to involve attachment to host cells or host-derived molecules (Bjarnsholt et al., 2009). In these instances it is challenging to extrapolate results obtained *in vitro* to chronic biofilm infections, as modifications that appear important for attachment to abiotic surfaces may be disposable for attachment to host tissues, or vice versa (Leuck et al., 2014). Furthermore, many biofilms encountered in the clinic are not comprised of a single species of bacteria, but rather contain mixtures that can include pathogenic or non-pathogenic bacteria of host or environmental origin, as well as fungi (Wargo and Hogan, 2006; Elias and Banin, 2012). These types of microbial interactions have been studied extensively in the CF lung, where *P. aeruginosa* has been shown to interact with the Bcc opportunistic pathogens *B. cepacia* and *B. cenocepacia* (Chattoraj et al., 2010; Schwab et al., 2014), as well as the pathogenic fungus *Aspergillus fumigatus* (Chotirmall and McElvaney, 2014). In the case of mixed *P. aeruginosa* and *B. cenocepacia* biofilms, the production of alginate by *P. aeruginosa* has been shown to promote *B. cenocepacia* persistence in a mouse model of CF (Chattoraj et al., 2010). This highlights how the production of EPS in mixed species biofilms can have implications that extend beyond the source organism, and suggests that data obtained regarding the presence or absence of EPS modifications in monospecies biofilms *in vitro* may have additional significance in multispecies biofilms.

Following bacterial culturing, difficulties in polysaccharide isolation due to the compositional complexity of the biofilm matrix or polymer insolubility may necessitate extensive optimization of purification protocols. Typically, separation of the polymer from the cell surface may require the use of procedures that lyse the associated bacteria, introducing additional contaminants (Bales et al., 2013). Therefore, it is preferable if possible to isolate EPS from culture supernatants; however, such polymers may exhibit different properties from their cell-associated counterparts leading to discrepant analysis (Maira-Litràn et al., 2002). In either case, once the polymer has been obtained from the cell surface or supernatant, contaminants such as DNA, RNA and protein must be removed. This can be achieved through enzymatic digestion or chemical precipitation of contaminants, or precipitation of EPS. Contaminating carbohydrates can be removed by chromatographic techniques such as size exclusion or ion exchange (Bales et al., 2013). In some cases, purification of EPS leads to insufficient yields for subsequent compositional or structural analysis, often as a result of polymer insolubility. As a result, harsher conditions may need to be employed to solubilize the polymer, including the use of strong acids or bases. During initial structural studies of PIA, strong alkaline purification conditions led to the incorrect identification of *N*-succinyl groups on the polymer (McKenney et al., 1999; Maira-Litràn et al., 2002), which was actually a degradation product of glucosamine monosaccharides (Sadovskaya et al., 2005). Additionally, phosphates have been reported in the monosaccharide composition of polymers, which are occasionally remnants from purification buffers, or teichoic acids in the case of Gram-positive bacteria (Sadovskaya et al., 2005). Harsher conditions can also partially or completely remove functional groups, such as acetyls, or even degrade the polymer, leading to incorrect calculations of molecular weight. The EPS described in this review are the select few whose structures have been determined, or for which we have biochemical and genetic data supporting the importance of the polysaccharide modifications. With improved culturing, purification and structural determination procedures, additional EPS will be discovered and their modifications characterized. We will then be able to formulate trends between different types of modifications and their effect on biofilm formation, pathogenicity, and virulence. Furthermore, the details regarding the genes and proteins involved in the addition of polysaccharide modifications remain largely unavailable. Characterization of these genes and proteins will likely provide details on how the levels and types of modifications are regulated under different conditions. Such findings will have a significant impact on our understanding of bacterial pathogenicity, and may reveal novel drug targets aimed at inhibiting the biosynthesis of these important virulence factors.

## Conflict of Interest Statement

The authors declare that the research was conducted in the absence of any commercial or financial relationships that could be construed as a potential conflict of interest.

## Abbreviations

Bcc: *Burkholderia cepacia* complex
CF: cystic fibrosis
CPS: capsular polysaccharide
EPS: exopolysaccharide
G-blocks: guluronate blocks
GAGs: glycosaminoglycans
GBS: Group B *Streptococcus*
GlcA: glucuronic acid
GulA: guluronate
GulNAcA: *N*-acetyl guluronate
HS: heparan sulfate
IdoA: iduronic acid
LPS: lipopolysaccharide
M-blocks: mannuronate blocks
ManA: mannuronate
MBOAT: membrane-bound *O*-acetyltransferase
MG-blocks: mixed mannuronate and guluronate blocks
PEL: pel polysaccharide
PIA: polysaccharide intercellular adhesin
PNAG: poly-*N*-acetylglucosamine
polyM: polymannuronate
ROS: reactive oxygen species
VPS: *Vibrio* polysaccharide.
